# Designed Antimicrobial Peptides for Topical Treatment of Antibiotic Resistant Acne Vulgaris

**DOI:** 10.3390/antibiotics9010023

**Published:** 2020-01-13

**Authors:** Kathryn W. Woodburn, Jesse Jaynes, L. Edward Clemens

**Affiliations:** 1 Riptide Bioscience, Inc., 941 Railroad Avenue, Vallejo, CA 94952, USA; eclemens@riptidebio.com; 2 Integrative Biosciences, College of Agriculture, Environment and Nutritional Sciences, Tuskegee University, Tuskegee, AL 36088, USA; jjsqrd@bellsouth.net

**Keywords:** acne, antimicrobial peptides, bacterial resistance, multidrug resistance, topical treatment

## Abstract

Acne vulgaris, caused by the Gram-positive bacterium *Cutibacterium acnes*, is a prevalent dermatologic condition with substantial cutaneous and psychological morbidity. Mild acne is treated with topical antibiotics with more severe inflammatory forms requiring the prolonged use of oral antibiotics, resulting in antimicrobial resistance development. Innovative treatment alternatives, providing complete microbicidal eradication with minimal safety issues and limited susceptibility to microbial resistance, are fervently sought. Designed antimicrobial peptides (dAMPs) are engineered analogs of naturally occurring AMPs that possess a reduced likelihood of developing bacterial resistance. Seven novel dAMP sequences were screened for in vitro bactericidal effectiveness against antibiotic resistant *C. acnes* clinical isolates. Five peptides (RP444, RP551, RP554, RP556, and RP557) exhibited potent in vitro antibacterial activity. The Therapeutic Index, a measure of specificity for killing multidrug resistant *C. acnes* over mammalian cells, was determined using bioluminescent human keratinocytes. The Therapeutic Index was highest for the disulfide dAMP, RP556, with a value of 130. The lead dAMP candidate RP556, was further evaluated in a multidrug-resistant *C. acnes* intradermal murine infection model. A topical application of 5 mg/mL RP556 (0.5%) eliminated infection. If these preclinical results are translated clinically, dAMPs may become a viable topical monotherapy for the treatment of recalcitrant acne infections.

## 1. Introduction

Acne vulgaris is a chronic inflammatory skin disorder affecting more than 80% of all adolescents and young adults worldwide [1]. The disease can manifest severe social and psychological expressions, including crippling effects on patients’ self-esteem and socialization. The pathogenesis of acne is multifactorial and usually implicates follicular colonization with the Gram-positive *Cutibacterium acnes* (*C. acnes*; formerly *Propionibacterium acnes*), resulting in bacterial overgrowth and inflammation. Current acne treatments present side effects such as erythema, scaling, burning, hair bleaching [2], bacterial resistance, and teratogenic effects [3]. Mild forms of acne are often treated with topical antibiotics, with severe inflammatory forms requiring the prolonged use of oral antibiotics resulting in the development and spread of antimicrobial resistance [4]. Many countries report that more than 50% of *C. acnes* strains are resistant to antibiotics, making them substantially less effective [1].

Monotherapy for the management of acne with currently approved topical antibiotics [5], according to the American Academy of Dermatology “Guidelines of Care for the Management of Acne Vulgaris,” is not recommended due to concerns related to the development of antibiotic resistance. Clindamycin is the preferred topical antibiotic; however, its widespread and often permissive use has led to the generation of resistant strains [5]. Topical erythromycin is also used; however, it has reduced efficacy in comparison with clindamycin, and *C. acnes* exhibits resistance to it. Moreover, *Staphylococci* have generated resistance following treatment of *C. acnes* patients [6]. In an attempt to reduce the development of bacterial resistance, systemic antibiotic treatment is limited to 3 months as recommended by the American Academy of Dermatology [5]. Despite the critical need for new antibiotics with novel modes of action that are active against multidrug resistant (MDR) pathogens, the development of antibacterial agents has drastically declined [7]. Therefore, a monotherapy acne treatment that is highly potent with a reduced likelihood of developing antimicrobial resistance and with anti-inflammatory activity is urgently needed.

Designed antimicrobial peptides (dAMPs) are laboratory synthesized peptides that are rationally designed analogs of naturally occurring AMPs, which provide the first line of defense against invading pathogens in all multicellular organisms [8,9,10]. dAMPs, also known as host defense peptides, have direct antibiotic activities in addition to modulating immune responses [8]. dAMPs possess an amphipathic α-helix or β-sheet structure and a net positive charge, physicochemical features crucial as the peptides act as antimicrobial agents by electrostatically interacting and selectively perturbing the barrier function of the bacterial membrane. This membrane is rich in anionic phospholipids and negatively charged lipopolysaccharides, in contrast to mammalian cells that are predominantly composed of zwitterionic phospholipids [11]. The remarkable targeting and direct disruption of the bacterial membrane makes bacterial resistance development less likely [8].

## 2. Results

### 2.1. Designed Antimicrobial Peptides

**Figure 1:**
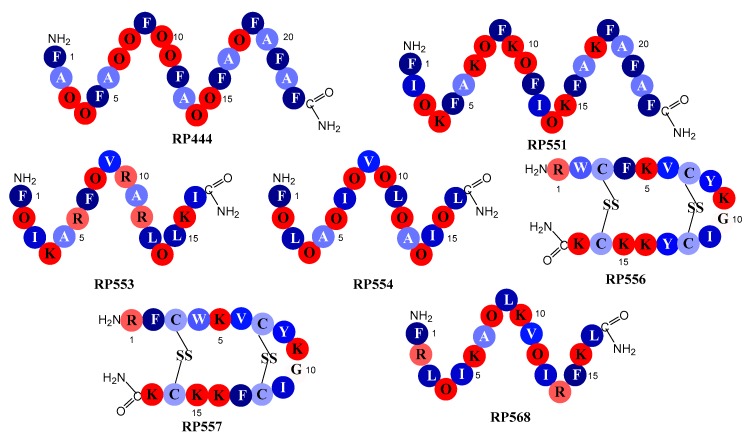
Schematic representation of the designed antimicrobial peptides. Single letter codes of the amino acids are depicted with color coding representing relative hydrophobicity. All hydrophobic amino acids are colored blue while hydrophilic amino acids are red. The number values are normalized and relative hydrophobicities are represented by the number of kcal/mole necessary to move an amino acid in an α-helix from the aqueous phase to the inside of a lipid bilayer [13]: F, phenylalanine, −3.85; L, leucine, −3.36; I, isoleucine, −3.16; Y, tyrosine, −2.66; V, valine, −2.34; W, tryptophan, −1.96; A, alanine, −1.56; C, cysteine, −1.06; G, glycine, −0.14; R, arginine, 2.22; O, ornithine, 3.56; and lysine, K, 3.85.

The seven dAMPs evaluated here, whose amino acid sequences are outlined in Figure 1, were carefully tailored to improve upon both natural and synthetic AMP libraries. Iterative evaluations to obtain optimal anti-pathogenic function have found that acceptable activity is attained when the peptide contains an amphipathic region of around 17 contiguous amino acids. To optimize the inhibition of pathogenic activity, while concurrently minimizing eukaryotic toxicity, the hydrophobicity of the non-polar region was maximized by using phenylalanine (F), isoleucine (I), and leucine (L). These conformational changes are coupled, in an effort to maximize the positive charge density of the peptide’s polar region, by the incorporation of lysine (K) and or/arginine (R) and C-terminus amidation. The replacement of lysine (K) with the non-natural amino acid, ornithine (O), was made to increase antibacterial activity while also enhancing proteolytic stability [12].

### 2.2. C. acnes In Vitro Antibacterial Activity

Seven novel dAMP sequences (Figure 1) were screened for their in vitro bactericidal effectiveness; Minimum Inhibitory Concentration (MIC) with dosing range between 0.5 µg/mL and 32 µg/mL, against ten *C. acnes* clinical isolates from BEI resources and two *C. acnes* ATCC reference strains. RP444, RP551, RP554, RP556 and RP557 were the most active peptides against the *C. acnes* bacterial strains, while RP553 showed modest anti-bacterial activity and RP568 exhibited negligible activity over the 0.5–32 μg/mL concentration range evaluated (Table 1). The dAMPs exhibited similar activity against all *C. acnes* isolates evaluated, including antibiotic-resistant *C. acnes* strains (tetracycline, erythromycin and clindamycin).

### 2.3. Limited Mammalian Cytotoxicity

**Figure 2:**
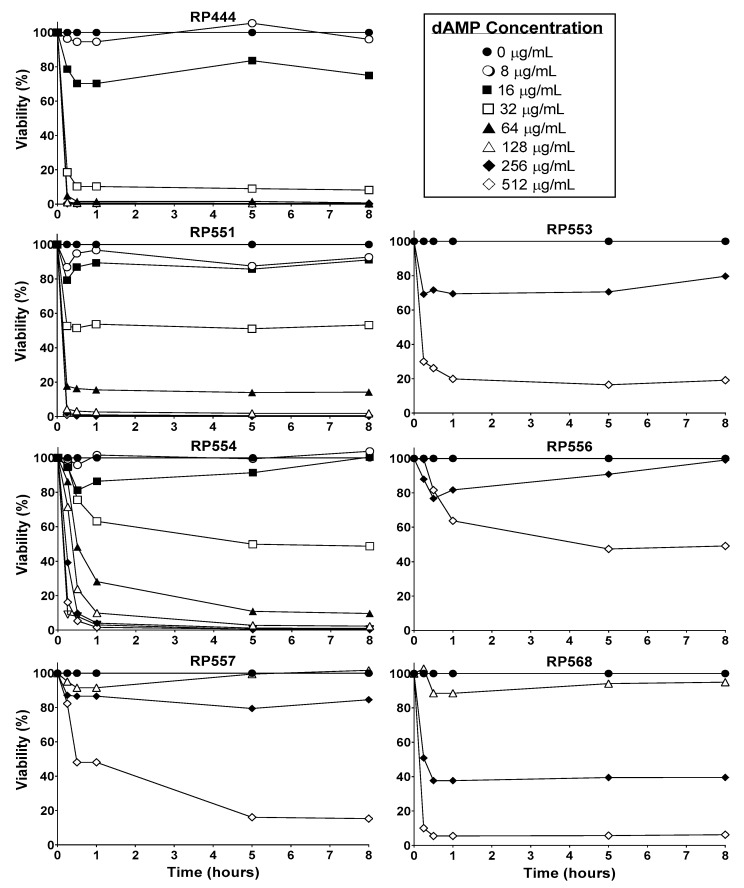
dAMPs, RP553, RP556, RP557 & RP568, exhibit minimal eukaryotic toxicity. Temporal and dose-response curves of human keratinocyte cytotoxicity through 8 h. Cells were plated at 1 × 10^4^ cells/well, allowed to adhere overnight, the specific dAMP was added, and cytotoxicity evaluated through 8 h. Cellular toxicity was noninvasively assayed using a bioluminescent strain of human keratinocytes and viability assayed using an IVIS Lumina imaging system (Perkin Elmer). Data shown represent the mean of triplicate replicates.

The candidate dAMPs, based on physicochemical properties, preferentially destroy bacterial membranes over mammalian membranes. In order to confirm this activity, the time and concentration-dependent effects of dAMPs on human keratinocytes were evaluated (Figure 2). Limited toxicity to mammalian cells was observed after 8 h of exposure with RP553, RP556 and RP557, all of which showed at least greater than 80% viability following 8 h incubation at 256 μg/mL, with RP556 exhibiting the least amount of cytotoxicity.

### 2.4. High Therapeutic Index

**Figure 3:**
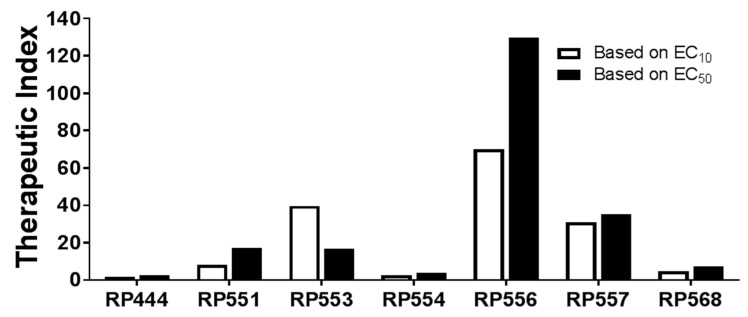
RP556, RP557 and RP553 possess potent selectivity for *C. acnes* compared to human keratinocytes. dAMP Therapeutic Index evaluation defined by the dose required to kill 10% (EC_10_) and 50% (E_C50_) of human keratinocytes compared to the *C. acnes* minimal inhibition concentration (MIC).

In evaluating the clinical utility of each of the candidate dAMPs for *C. acnes* treatment, the Therapeutic Index was determined. The Therapeutic Index is defined as the ratio of concentration at which the dAMP is active against the prokaryotic *C. acnes* pathogen without inducing cytotoxic damage to the surrounding mammalian cells. The EC_10_ and EC_50_ doses, defined as the concentrations required to kill 10% and 50% respectively of human keratinocytes following 8 h incubation with each of the Riptide dAMPs, are tabulated and graphed in Table 2 and Figure 3, respectively. The larger the Therapeutic Index, the greater the specificity of the dAMP for destroying *C. acnes* cells.

RP556 displayed the highest Therapeutic Index with ratios of 130 and 70 for EC_10_ and EC_50_, respectively. RP557 and RP553 yielded similar ratios with values of 35 and 16.8, respectively for EC_50_ evaluations. RP444, RP554, and RP568 yielded the lowest cell selectivity ratios with EC_50_ index values of 2.5, 3.88, and 7.16, respectively.

### 2.5. RP556 Activity in a Multidrug Resistant C. acnes Murine Model

**Figure 4:**
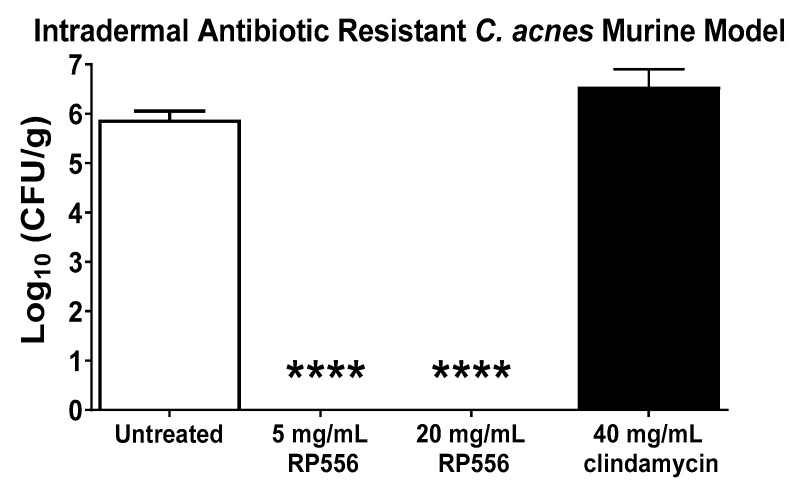
RP556 eradicates *C. acnes* infection. RP556 (5 or 20 mg/mL) or 40 mg/mL clindamycin was applied topically (using a volume of 25 μL) 2, 14, 26, 38, 50, 62 and 74 h post infection for a total of 7 applications to BALB/c mice infected with antibiotic-resistant *C. acnes*. Skin was harvested at 96 h for bacterial CFU/gram of skin tissue. Each data point represents the mean ± SE of six measurements while clindamycin treatment represents four data points. RP556 at 5 and 20 mg/mL are statistically significant compared to both untreated and clindamycin treated animals (****, *p* < 0.0001) using a one-way ANOVA followed by a Dunnett’s post hoc comparison test.

A multidrug resistant dermal murine model was developed to evaluate the effectiveness of the lead dAMP, RP556. HL043PA2 is resistant against clindamycin, tetracycline and erythromycin [14]. At 96 h post-infection, untreated mice demonstrated a mean bacterial burden of 6 log10 CFU/g (Figure 4). There were no untoward clinical observations or changes in body weight following treatment. All mice topically treated with 25 μL applications of RP556 (both 5 and 20 mg/mL) had mean bacterial burdens below the detectable limit, representing statistically significant decreases compared to both untreated and clindamycin-treated animals.

## 3. Discussion

Acne vulgaris is a common skin disorder prevalent among adolescents and young adults [5]. Acne vulgaris can have a profound cutaneous and psychologic disease burden, leading to anxiety, low self-esteem and depression. Significant antibiotic resistance and multiple drug resistance have been observed for *C. acnes* strains from acne patients following long-term antibiotic treatments [1]. The increase in antibiotic resistant *C. acnes* infections has generated an urgent need for new antibacterial agents with novel modes of action and an inherent limited likelihood of developing resistance.

Designed antimicrobial peptides (dAMPs) are engineered analogs of naturally occurring AMPs, which are ubiquitous in nature and provide the first line of defense against invading pathogens. Seven novel dAMPs were synthesized and evaluated for activity against ten antibiotic resistant *C. acnes* and two ATTC reference strains. Five dAMPs (RP444, RP551, RP554, RP556 and RP557) exhibited potent activity against all *C. acnes* isolates with MIC values of 2–8 μg/mL.

An efficacious antimicrobial must be able to selectively inhibit and kill bacteria. The development of a clinically viable dAMP has been hampered by unwanted toxicity to mammalian host cells at therapeutic doses [15]. Therefore, the cytotoxicity of dAMPs was evaluated in human keratinocytes. The in vitro cytotoxicity of dAMPs on human keratinocytes was determined using a newly developed bioluminescence assay. At doses of dAMPs that kill *C. acnes* upon contact, limited toxicity to eukaryotic cells after 8 h of exposure was observed, especially with RP553, RP556 and RP557, all of which showed at least greater than 80% viability following 8 h incubation at 256 μg/mL.

The Therapeutic Index, defined as the concentration at which the dAMP is active against the prokaryotic *C. acnes* pathogen without inducing cytotoxic damage (evaluated using both EC_10_ and EC_50_) to the surrounding mammalian cells, was evaluated to determine the clinical utility of the candidate dAMPs. RP556 and RP557 demonstrated the largest therapeutic indices with values of 130 and 35.1, respectively. RP556 was then evaluated in vivo, and to ensure the most clinically representative model was utilized, a multidrug-resistant *C. acnes*-induced dermal inflammation model was developed using the HL043PA2 *C. acnes* strain. 

Herein is the identification of a topical anti-infective that will inherently possess fewer adverse effects and drug interactions than systemic agents. RP556 completely eliminated *C. acnes* infection following a topical application of 5 mg/mL in a multidrug resistant murine dermal infection model. RP556 exhibits potent activity against *C. acnes* antibiotic-resistant strains, possesses a novel mode-of-action with a reduced likelihood of developing bacterial resistance, selectively targets *C. acnes* and not mammalian cells, and eliminates infection in a multidrug-resistant *C. acnes* intradermal murine infection model. If these results are clinically translated, dAMPs (specifically RP556) may serve as a monotherapy for recalcitrant *C. acnes* infection.

## 4. Materials and Methods

### 4.1. Designed Antimicrobial Peptides

Seven dAMPs, whose amino acid sequences are outlined in Table 1, were synthesized via solid phase synthesis (AmbioPharm, North Augusta, SC, USA). Peptide purity was >96% as assayed by high performance liquid chromatography and mass spectroscopy.

### 4.2. Minimum Inhibitory Concentration (MIC)

The ten antibiotic resistant *C. acnes* clinical isolates were acquired from BEI resources with the two reference strains obtained from American Type Culture Collection (ATCC, Manassas, VA, USA). *C. acnes* is a relatively slow-growing, typically aerotolerant anaerobic so all in vitro *C. acnes* experiments were performed under anaerobic conditions.

Single colonies were inoculated in Reinforced Clostridium Medium (RCM) broth supplemented with sodium thioglycolate and incubated at 37 °C, with shaking at 200 rpm, for 2 or 3 days to reach logarithmic growth (OD_600nm_ = 1.0). Each *C. acnes* (1 × 10^6^ CFU per mL) was incubated with dAMP or clindamycin at two-fold serial dilutions (0.5–32 μg per mL) in RCM on a 96-well microplate for 48 h. Three replicas were included for each test article concentration. Bacterial growth was measured by optical density at 600 nm to determine the MIC for each dAMP applied.

### 4.3. Mammalian Cytotoxicity

Noninvasive and real-time monitoring of mammalian cytotoxicity to assess the potential skin toxicity of the dAMPs was evaluated using bioluminescent human immortalized keratinocytes (HaCaT, AddexBio, San Diego, CA, USA). The bioluminescent variant of HaCaT was constructed by transfection with a luciferase gene (RediFect Red-Fluc-Puromycin, Cat# CL596002) [16]. Keratinocytes (1 × 10^4^ cells, 100 μL) were plated in 96-well black-walled plates. The candidate dAMPs were 2-fold serially diluted from 1024 µg/mL in growth medium supplemented with 150 µg/mL D-luciferin. Each concentration was performed in triplicate, with the final volume being 200 μL. Imaging was performed at select times (0, 15, and 30 min and 1, 3, 5, and 8 h) after the addition of the dAMP, and compared to concurrently run vehicle-control, using an IVIS Lumina imaging system (Caliper Life Sciences, Inc., Hopkinton, MA, USA). For imaging, the 96-well plate was positioned on the stage (12.5-cm field of view), with an open emission filter, binning of 4, and f-stop 1 and a 1-min exposure time. Data analysis was performed using the Living Image software program (version 4.3, Caliper Life Sciences, Inc.). The dAMP concentrations required to kill 10% and 50% of keratinocytes were determined using GraphPad Prism 7 (GraphPad Software, San Diego, CA, USA).

### 4.4. Animals

All animals received care in compliance with the Guide for the Care and Use of Laboratory Animals (8th Edition, National Institutes of Health Publication, 2011). All studies were approved by the Institutional Animal Care and Use Committee. Female BALB/c mice, 5 to 7 weeks, were obtained from Envigo Laboratories Inc (Indianapolis, IN, USA). General anesthesia consisted of an intraperitoneal injection of ketamine 100 mg/kg and xylazine 10 mg/kg (Vedco, Inc., St. Joseph, MO, USA).

### 4.5. In Vivo Efficacy Assessment of RP556 against Multidrug-Resistant HL043PA2 C. acnes

Mice were inoculated via intradermal (ID) injection of the antibiotic-resistant *C. acnes* HL043PA2 (7.0 log10 CFU, 50 μL, BEI Resources, HM-514, [14]). The day prior to infection, each mouse was anesthetized using isoflurane, and an area of approximately 1″ × 1″ of skin on the dorsal area of each mouse was shaved and cleared of hair using the depilatory agent Nair^®^. Beginning 2 h after infection, mice were anesthetized and RP556, formulated in 2% hydroxypropyl methylcellulose in water, viscosity 3000–5600 cP, or 40 mg/mL clindamycin (4%) was administered topically, at 25 μL twice daily for a total of 7 applications (2, 14, 26, 38, 50, 62 and 74 h post infection). Abscesses were harvested at 96 h post-challenge (or Day 7 for untreated control) and assessed for bacterial CFU per gram of skin tissue.

### 4.6. Statistical Analysis

Quantitative data were expressed as mean ± standard error of the mean. Statistical analysis was performed using GraphPad Prism 7 (GraphPad Software, San Diego, CA, USA). Comparisons were performed using a 1-way analysis of variance (ANOVA) followed by a post hoc Dunnett’s test. A *p* value <0.05 was considered statistically significant.

## Figures and Tables

**Table 1:** Growth Inhibition of dAMPs (MICs, µg/mL) against *C. acnes* Isolates.

*C. acnes*	Antibiotic Sensitivity			dAMPs						
	Tetra ^#^	Ery ^#^	Clind ^#^	RP444	RP551	RP553	RP554	RP556	RP557	RP568
ATCC6919	S	S	S	2	2	8	4	4	8	>32
ATCC11827	S	S	S	8	2–4	16	8	8	8	>32
HL007PA1	R	R	R	4	2	16	4	4	8	>32
HL013PA1	R	R	R	4	2	8	2–4	2	2	>32
HL038PA1	R	R	R	4	2	16	8	4	8	>32
HL043PA1	R	R	R	4	2	16	4	4	8	>32
HL043PA2	R	R	R	8	2	16	8	4	8	>32
HL045PA1	R	R	R	4	2	16	4	4	4	>32
HL053PA1	R	R	R	4	2	16	4	4	4	>32
HL056PA1	R	R	R	4	2	16	4	2	4	>32
HL072PA1	R	R	R	8	2–4	16	8	8	8	>32
HL082PA2	R	R	S	2	2	16	8	2	2	>32

Antibiotic sensitivity; S, sensitive; R, resistance. ^#^ Antibiotic resistance to clindamycin defined by MIC ≥ 32 μg/mL with resistance to tetracycline (Tetra), MIC ≥ 1.0 μg/mL and erythromycin resistance MIC ≥ 0.5 μg/mL [12]. Data represents the mean of three replicates.

**Table 2:** dAMP Therapeutic Index Evaluation.

dAMP	MIC (μg/mL)	EC_10_ (μg/mL)	Therapeutic Index	EC_50_ (μg/mL)	Therapeutic Index
RP444	8	13	1.63	20	2.5
RP551	2	16.6	8.3	34.3	17.2
RP553	16	238	39.7	268	16.8
RP554	8	19	2.38	31	3.88
RP556	4	276	70	519	130
RP557	8	246	30.8	281	35.1
RP568	>32	146	4.56	229	7.16

MIC values against the HL043PA2 multi-drug resistant *C. acnes* strain. EC_10_ and EC_50_ values were determined using the GraphPad Prism 7 program.
